# The Role of Toll-Like Receptors in Autoimmune Diseases through Failure of the Self-Recognition Mechanism

**DOI:** 10.1155/2017/8391230

**Published:** 2017-05-03

**Authors:** Mark Farrugia, Byron Baron

**Affiliations:** Centre for Molecular Medicine and Biobanking, Faculty of Medicine and Surgery, University of Malta, Msida MSD 2080, Malta

## Abstract

Toll-like receptors (TLRs), part of the innate immune system that recognises molecular signatures, are important in the recognition of pathogenic components. However, when specific cellular contexts develop in which TLRs are inappropriately activated by self-components, this may lead to sterile inflammation and result in the occurrence of autoimmunity. This review analyses the available data regarding TLR biochemistry, the specific mechanisms which are brought about by TLR activation, and the importance of these mechanisms in the light of any existing and potential therapies in the field of autoimmunity.

## 1. Introduction

The innate immune system relies on a limited number of germline-encoded receptors, known as pattern recognition receptors (PRRs), that have evolved to recognise molecular signatures [1]. Toll-like receptors (TLRs) are just one of several distinct classes of PRRs including Nod-like receptors (NLRs), C-type lectin receptors (CLRs), AIM2-like receptors (ALRs), RIG-I-like receptors (RLRs), and intracellular DNA sensors including cyclic GMP-AMP synthase (cGAS) [2, 3], which recognise structures as diverse as flagellins, nucleic acids, saccharides (mainly mannose and lipopolysaccharide), peptidoglycans (such as lipoteichoic peptidoglycans), and lipoproteins. An adaptive immune response is triggered by the recognition of such antigens, mediated by proinflammatory cytokine production together with antigen-presenting cell (APC) stimulation.

TLRs are a family of type I transmembrane glycoproteins [4] consisting of a single transmembrane helix, which connects an extracellular ligand-binding domain to an intracellular signalling domain [5]. The extracellular domain can bind either directly to the ligand or to coreceptor-ligand complexes, and it then initiates ligand-mediated multimerisation of the receptor. TLRs generally are found as dimers, with most being homodimers, although TLR2 can be found preferentially as heterodimers either with TLR1 or with TLR6, even when a ligand is absent [6]. The intracellular signalling domains of TLRs have significant sequence similarity with the interleukin-1 receptor (IL-1) and are thus termed Toll/IL-1R homology (TIR) domains [4]. TLRs are located either at the cell surface or in the endosomes. TLR1, TLR2, TLR4, TLR5, and TLR6 are expressed on the cell membrane, whereas TLR3, TLR7, TLR8, and TLR9 are localised mainly in the endosomal compartment [7]. TLR expression has been identified in various immune cells, including T-cells, B-cells, different subsets of dendritic cells, and macrophages [8, 9].

The TLR family can recognise a wide variety of bacterial, fungal, protozoan, and viral components, generally referred to as pathogen-associated molecular patterns (PAMPs). These TLR ligands can be grouped into three categories: lipids and lipopeptides (recognised by TLR2/TLR1, TLR2/TLR6, and TLR4), proteins (recognised by TLR5), and nucleic acids (recognised by TLR3, TLR7, TLR8, and TLR9). Different types of nucleic acid have their unique TLR, with viral double-stranded RNA (dsRNA) being recognised by TLR3, single-stranded RNA (ssRNA) being recognised by TLR7 and TLR8, and DNA containing unmethylated CG dinucleotides (whether from bacteria, viruses, or synthetic oligodeoxynucleotides, referred to as ODNs) being recognised by TLR9 [1, 10, 11].

TLRs play an important role in both innate and acquired immune responses [4, 12, 13]. However, the inappropriate TLR activation triggered by self-components brings about sterile inflammation and autoimmunity. Autoimmunity is the result of several mechanisms that are associated with the presence of autoreactive immune cell subsets and loss of immunological tolerance [9]. Organ-specific autoimmune diseases are the culmination of hereditary and environmental factors related to the failure of adaptive immune response regulation to self-antigens [14]. In fact, overexpression of PRRs was identified in the tissues of patients with organ-specific autoimmunity, type 1 diabetes and Crohn's disease [15, 16].

Autoimmunity and infection have been linked together in several studies due to PAMPs being found in tissues after episodes of infection [17]. These are part of a substantial body of experimental data indicating that PRR activation on innate immune cells by either PAMPs or pathogens has the ability to dysregulate self-tolerance and subsequently activate autoreactive T- and B-cells.

However, TLRs are also known to recognise host-derived endogenous ligands which have undergone some form of change from their native state or accumulated excessively in nonphysiologic compartments [18]. Such ligands are referred to as damage associated molecular patterns (DAMPs) and are commonly released from damaged tissues or apoptotic cells such as high mobility group box 1 (HMGB1), saturated fatty acids, and amyloid *β* and can result in chronic or acute inflammation [19–21]. However, under certain conditions TLRs can bind to such kind of self-molecules and as a result contribute to the development, progression, and resolution of autoimmune diseases [22]. A list of potential endogenous ligands and their respective TLRs can be found in Table 1.

In order to prevent TLR-induced inflammation, TLR-activated DCs cross-talk with and are suppressed by regulatory T-cells (T_regs_), while the suppressive action of regulatory T-cells is blocked by APCs which have been stimulated through TLRs [23]. TLR stimulation can conversely be inhibited by cytokines such as interleukin- (IL-) 10 secreted by regulatory T-cells [24, 25]. Different subsets of T_regs_ exist and their impairment has been reported in a number of human autoimmune conditions, including oral tolerance [26].

## 2. TLR Signalling Pathways

**Figure 1:**
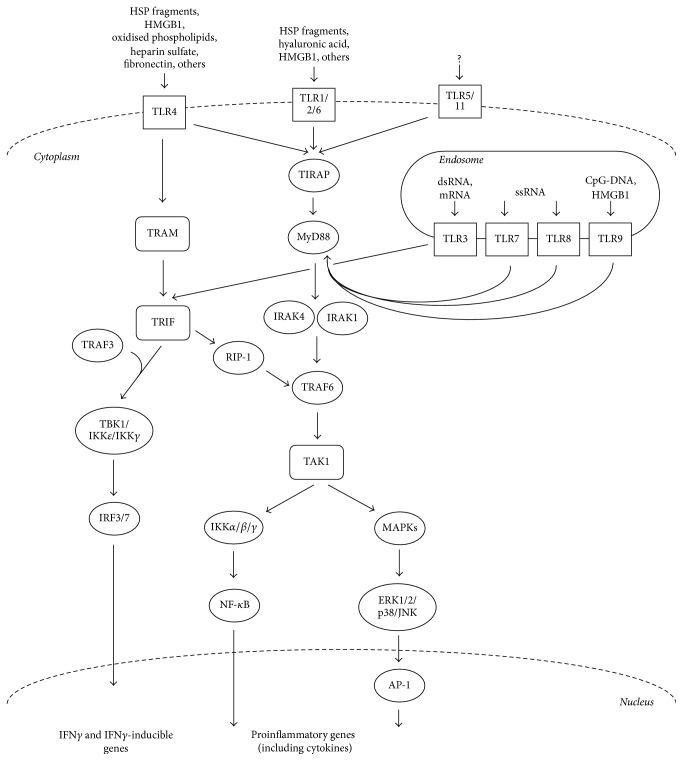
TLR signalling pathways. The two main pathways by which TLR signalling occurs upon stimulation by their ligands. These pathways are characterised depending on which of the adaptor molecules MyD88 or TRIF are involved. These signalling cascades result in the activation of NF-kB, IRFs, and MAP kinases, which promote the transcription of various immune response genes, including inflammatory cytokines and IFN*γ*-related genes. (AP-1, activator protein 1; ERK, extracellular signal-regulated kinase; IKK, inhibitor of kappa light polypeptide gene enhancer in B-cell kinase; IRAK, IL-1 receptor-associated kinase; JNK, c-Jun N-terminal kinase; MKK, MAPK kinase; RIP1, receptor interacting protein 1; TAK, transforming growth factor-activated kinase; TBK1, TANK-binding kinase 1; TRAF, tumour necrosis factor receptor-associated factor.)

The stimulation of TLRs by the respective ligands results in the recruitment of downstream adaptor molecules, such as myeloid differentiation factor 88 (MyD88), myeloid Toll/interleukin- (IL-) 1 receptor- (TIR-) domain-containing adaptor-inducing interferon-*β* (TRIF a.k.a. TIR-domain-containing molecule 1; TICAM1), TIR-associated protein (TIRAP a.k.a. MyD88 adaptor-like; Mal), TRIF-related adaptor molecule (TRAM), or sterile *α*- and armadillo-motif containing protein (SARM) [6, 27, 28], which trigger a number of downstream molecules activating signalling cascades converging at the nuclear factor-kB (NF-kB), interferon (IFN) response factors (IRFs), and mitogen-activated protein (MAP) kinases. These transcription factors induce the transcription of various immune response genes, including inflammatory cytokines (such as interleukin- (IL-) 6, IL-12, IL-23, and tumour necrosis factor *α* (TNF-*α*)), stimulatory immune cytokines, chemokines, and costimulatory molecules [29, 30] (Figure 1).

TLRs specifically recruit and signal via TRIF, TIRAP, TRAM, or direct MyD88 activation to produce a different response, despite this recruitment being always through heterotypic TIR-TIR interactions. More specifically, except for TLR3, MyD88 is utilised by all TLRs and activates NF-kB and mitogen-activated protein kinases (MAPKs) to induce the genes encoding inflammatory cytokines [31]. TIRAP acts as a sorting adaptor by recruiting MyD88 to TLRs at the cell surface. One study has also shown that TIRAP is involved in endosomal TLR signalling (such as TLR9) by binding to different lipids [32, 33]. The recruitment of TRIF to TLR3 and TLR4 promotes an alternative pathway to that of MyD88 [34]. Interestingly, TRAM has been shown to be recruited specifically to TLR4 but not TLR3 and serves as the link between TRIF and TLR4 [35]. Thus, depending on what adaptor protein is used, one can divide TLR signalling into two pathways: MyD88-dependent and TRIF-dependent pathways.

In the MyD88-dependent pathway, TLR binding leads to MyD88 forming a complex with IRAK kinases [36], such that IRAK4 activates IRAK1 [37, 38]. IRAK1 then associates with TRAF6, a RING-domain E3 ubiquitin ligase, which polyubiquitinates and activates TAK1 [39, 40]. This results in the activation of the NF-kB and MAPK pathways. NF-kB is activated through the IKK complex (composed of the IKK*α* and IKK*β* catalytic subunits and the IKK*γ* regulatory subunit), which phosphorylates the NF-kB inhibitory protein IkB*α*, allowing the translocation of NF-kB to the nucleus inducing the expression of proinflammatory genes. The MAPK pathways are induced through ERK1/2, p38, and JNK, which mediate activation of AP-1 and regulate inflammatory responses [2, 41].

In the TRIF-dependent pathway, once TRIF is activated by either TLR3 or TLR4, it binds to TRAF6 via the recruitment of RIP-1 kinase, which leads to the activation of TAK1. This results in the NF-kB and MAPKs induction of inflammatory cytokines as discussed above. Meanwhile, TRIF also activates TRAF3 which recruits IKK-related kinases (TBK1 and IKK*ε*) and IKK*γ* for IRF3 phosphorylation, which translocates into the nucleus inducing the expression of type I IFN genes [2, 41]. On the other hand, TRAF3 binds to the MyD88 complex causing its degradation and leading to TAK1 activation. This implies that TRAF3 promotes the TRIF-dependent pathway via direct activation, while concurrently inhibiting the MyD88-dependent pathway.

## 3. Nucleic-Acid-Sensing TLRs in Autoimmune Disease

The nucleic-acid-sensing TLRs are endosomal and consist of TLR3 (which recognises viral dsRNA [42]), TLR7 and TLR8 (which recognise viral ssRNA [43–45]), and TLR9 (which recognises bacterial and viral unmethylated CpG-containing DNA motifs [46]). TLR3 signalling is distinguished from the rest in that it signals via the adaptor TRIF, while TLR7, TLR8, and TLR9 signal through the adaptor molecule MyD88 [47]. TLR3 also binds to self-mRNA [48].

The restricted access to these TLRs is one mechanism by which to prevent them from being aberrantly activated by self-nucleic acids (as those released by apoptotic cells), which could bring about autoimmune diseases. However, the dysregulation of TLR7, TLR8, and TLR9 plays a major role in numerous autoimmune diseases.

Studies show that immune complexes (ICs) composed of DNA and the immunoglobulin G (IgG) that recognises them stimulate pDCs in humans to produce IFN*α* [11, 20, 49, 50] and the production of autoantibodies in mice via a process which involves TLR7 and/or TLR9 [51, 52]. Such ICs containing self-nucleic acids are recognised via the FcR*γ*IIa receptor expressed on the surface of plasmacytoid dendritic cells (pDCs; innate immune cells) [53]. Once antibodies are captured, the FcR allows ICs to enter pDC and activates the endosomal pathway [54]. In the endosome, the nucleic acid component of the IC is likely released by the low pH. The nucleic acids then trigger TLR7 or TLR9 which brings about the maturation of the pDC and induces IFN*α* production [50, 54, 55].

The trafficking of nucleic-acid-sensing TLRs from the endoplasmic reticulum to endosomes for ligand recognition as well as their nucleic acid-sensing function is controlled by the multipass transmembrane protein Unc93B1 [56]. This protein is also involved in the localisation regulation of nucleic-acid-sensing TLRs, with studies presenting evidence of competition between TLR7 and TLR9 for Unc93B1-dependent trafficking, where TLR9 predominates due to a higher affinity for Unc93B1 than TLR7 [56–59]. Thus, Unc93B1 regulates excessive TLR7 activation by balancing TLR9 to TLR7 trafficking. The mutation of Unc93B1 by an amino acid substitution (D34A) resulted in a phenotype which presents TLR7-hyperreactivity together with TLR9-hyporeactivity, which was linked to systemic lethal inflammation, known to be TLR7-dependent [59]. It is thus hypothesised that in some autoimmune diseases deficiency of either TLR8 or TLR9 will result in decreased competition of these endosomal TLRs to bind to Unc93B1, making TLR7 more available and resulting in higher TLR7 trafficking and response. This manifests itself as autoimmune phenotypes [60]. Consequently, altering the TLR7 to TLR9 balance can be envisioned as a potential regulatory mechanism in the context of autoimmunity [59].

TLR7 activity regulation appears to be cell type dependent, with the TLR7 response being controlled by TLR8 on dendritic cells and by TLR9 on B-cells. This has been shown using the TLR7 agonist R848 on TLR8^−/−^ and TLR8/9^−/−^ derived from bone marrow (which produced significantly higher amounts of IFN*β* mRNA (at 4 h), IL-6, and TNF, compared with WT or TLR9^−/−^ cells) and TLR8/9^−/−^ and TLR9^−/−^ B-cells (which presented significant CD69 and CD86 upregulation, when compared to TLR8^−/−^ or WT B-cells) [60]. Concomitantly, TLR8^−/−^ and TLR8/9^−/−^ B-cells presented a significant increase in TLR7 mRNA levels compared to WT or TLR9^−/−^ cells. Furthermore, TLR8/9^−/−^ mice had a significantly higher percentage of activated and memory CD4 and CD8 T-cells compared to WT or TLR9^−/−^ mice, while in TLR8^−/−^ mice the values were only significantly higher when compared to WT mice [60].

## 4. Protein-Sensing TLRs in Autoimmune Disease

TLR2 and TLR4, which are both cell surface TLRs, are involved in regulating Th immune responses. In fact, it was shown that, depending on which TLRs are ligated, the Th responses can be skewed towards a Th1 or Th2 cytokine profile [61]. This is in contrast with a previous model in which Th2 differentiation was driven forward without any TLR-mediated signals. Despite the promotion of Th2 responses (and allergic inflammation) in some contexts by low-doses of inhaled LPS [62], generally TLR4 stimulated by LPS brings about the maturation of DCs associated with strong Th1-type responses as a consequence of IL-12 release [63]. Moreover, both TLR2 and TLR4 were found to be involved in regulating *γδ* T-cell [64] and CD4+ T-helper 17 (Th17) [65] production of IL-17. More specifically, in vitro studies showed that in *γδ* T-cells expansion and IL-17 production are promoted by the activation of TLR4 [65]. TLR2 signalling could also stimulate naïve cells to expand and undergo polarisation towards the Th17 lineage [65] and provide CD4^+^ and CD8^+^ T-cells with a costimulatory signal [66, 67].

TLR2 and TLR4 have been shown to also respond to endogenous heat-shock proteins [68, 69]. Clinical observations confirm that HSPs are involved in the regulation of some autoimmune disease such as autoimmune arthritis, type 1 diabetes, atherosclerosis, multiple sclerosis, and others [70].

## 5. Role of TLRs in Autoantibody Production

TLRs are known to play a critical role in autoantibody production because genetic mutations which lead to some deficiency in either TLR7, TLR9, or MyD88 most commonly also lead to a reduction in autoantibody production, while an increase in TLR7 production generally leads to a susceptibility towards developing autoimmune diseases [71]. Moreover, the engagement of B-cell receptor by chromatin-IgG complexes and TLR9 signalling were shown to be essential and act synergistically in the activation of autoreactive B-cells in vitro [51].

However, it is difficult to gauge the direct contribution of TLR signalling in B-cells in vivo since the activation of both DCs and plasmacytoid DCs is brought about by the same TLR ligands and play just as important roles in the pathogenesis of autoimmune diseases [20]. Furthermore, TLRs are expressed by a variety of cell types that have the ability to regulate the functions of B-cell through the activation of their TLRs. So far evidence seems to suggest that the intrinsic TLR signalling of B-cells is a requirement for the production of autoantibodies in vivo [72–74].

## 6. Spectrum of TLR Action in Autoimmune Disease Contexts

The involvement of TLRs in early inflammatory immune responses is responsible for their ability to develop autoreactive B- and T-cells. There is now a well-established link between a variety of infections and autoimmune diseases, both in animal models and in the clinical setting. Such autoimmune responses are thought to be the result of molecular mimicry of the pathogen-derived antigens to self-antigens or a form of nonspecific activation of the innate immune system (through loss of immunological tolerance) that results in the production of T-cells and antibody responses specific to self-antigens, implicating TLRs in numerous autoimmune diseases. Here we would like to point out three disease settings.

### 6.1. TLRs in Rheumatoid Arthritis

Several of the risk alleles linked to rheumatoid arthritis (RA) are associated with immune regulation such as NF-*κ*B-dependent signalling, T-cell stimulation, activation, and functional differentiation. This suggests that these immunologic pathways are amongst the key modulators of the development of the autoimmune inflammation in RA [75, 76]. Through the use of human synovial membrane cultures from patients with RA it has been shown that expression of TLR2 and TLR4 in conjunction with the release of endogenous ligands to these two TLRs may play a role in inflammation and damage to joints in RA patients [77]. The alarmin heterogeneous nuclear ribonucleoprotein (hnRNP) was also found to play a role in human RA and pristane-induced arthritis in rats. This RNA- and DNA-binding complex binds to most nuclear antigen-specific B and T-cells in systemic autoimmune diseases. Moreover hnRNP was shown to activate APCs causing arthritis mediated by TLR7 and TLR9, which can then activate harmful T-cells [78].

### 6.2. TLRs in Type 2 Diabetes Mellitus

Innate immune responses leading to Type 2 Diabetes Mellitus (T2DM) can be initiated by adipose tissue inflammation, as has been shown through LPS-treatment of adipocytes which showed significant increase in TLR2, TRAF-6, and NF-*κ*B levels as well as increased secretion of potentially diabetogenic proinflammatory cytokines. Similarly, abdominal subcutaneous adipose tissue isolated from diabetic patients presented increases in the expression of TLR2, MyD88, TRAF6, and NF-*κ*B. It has thus been proposed that obesity may increase proinflammatory cytokines in the host and subsequently increase the risk of T2DM [79]. TLR4 is thought to be an important mediator of obesity and insulin resistance because of its activation by saturated fatty acids and LPS, known to cause insulin resistance in adipose tissue amongst others. Obesity is associated with insulin resistance and is considered to be amongst the major risk factors in the development of T2DM and cardiovascular diseases. Thus, TLR4 is being considered as a potential therapeutic target in the treatment of these metabolic diseases [4].

### 6.3. TLRs in Other Diseases Not Generally Classified as Autoimmune

Two widespread diseases that are considered to have an autoimmune component are atherosclerosis and cancers. Both have now been shown to display TLR-directed responses. In the case of atherosclerosis a link has been found with TLR-induced IFN*α* [18, 80, 81], while, in cancer, the cells dying from chemotherapy release endogenous molecules that bring about an innate immune response following TLR-mediated recognition, which has been found to be critical for cancer elimination [82].

## 7. Therapeutic Application of TLR Regulators

TLR signalling is negatively regulated by a number of molecules, which act through a variety of mechanisms in order to prevent or control excessive immune responses. In autoimmunity and inflammatory diseases these processes could be nonfunctional, dysregulated, or simply not enough to overcome the TLR signalling in place. In an attempt to control aberrant immune responses both natural and synthetic negative regulators targeting each of the key molecules in the TLR signalling pathways have been identified. In fact, blocking the activation signal at the TLR level rather than targeting individual PAMPs or DAMPs is a more preferred and effective therapeutic approach since sterile inflammation contexts tend to be induced by similar signalling pathways and there may be multiple autoimmunity inducing peptides following tissue injury.

Cationic peptides (such as LL-37 or polymyxin), for example, are known to facilitate the uptake of self-DNA by plasmacytoid dendritic cells (pDCs) to activate TLR9 signalling and can lead to preferential localisation in the early endosomes of pDCs [81, 83], leading to significant IFN*α* production [83, 84]. One context in which this applies is psoriasis, in which the DNA is not transported in the form of an IC but associated with the antimicrobial cationic peptide, LL-37 [81]. Synthetic ligands of TLRs that effectively regulate innate and adaptive immune responses thus have the potential of being a new class of therapeutic molecules [85].

Some of these agents act directly on the TLRs themselves. Vitamin D3 has been shown to downregulate TLR2 and TLR4 expression on monocytes [86]. Endosomal TLRs like TLR7, TLR8, and TLR9 can be inhibited by Chloroquine and related compounds, which act as antagonists that specifically target and inhibit signalling through these TLRs [87–91]. Nucleic acid-binding polymers are another class of endosomal TLR inhibitors which recognise ssRNA, dsRNA, or hypomethylated DNA and bind to them, thus inhibiting nucleic acid-mediated activation of these TLRs [30]. 9-Benzyl-8-hydroxy-2-(2-methoxyethoxy) adenine on the other hand acts as a TLR7 agonist, which induces hyporesponsiveness or tolerance, thus still inhibiting TLR7 activation [92]. Research in such agonists could lead the way to the synthesis of drugs which could inhibit TLR overactivation while still sparing the side-effects related to total inhibition of these pathways.

At the protein level activation of the MyD88-dependent pathway is suppressed by the synthetic inhibitor ST2825, which is a heptapeptide analog specifically designed to inhibit MyD88 dimerisation, thus interfering with the recruitment of IRAK1 and IRAK4 by MyD88 [93]. Compound 4a is also responsible for inhibiting the MyD88 dependent pathway activation by interfering with the interaction between MyD88 and the TIR domains of TLRs [94]. Other inhibitors of MyD88 include Suppressor of Cytokine Signalling 1 (SOCS1) and the E3 Ubiquitin Protein Ligase Cbl-b. Further down the pathway IRAK1 and IRAK4 are targeted by RO0884 which acts as a dual inhibitor of both these protein kinases resulting in the blocking of proinflammatory cytokine production [95]. TRAF6 is targeted by a number of inhibitory molecules such as Tumour Necrosis Factor Inducible Protein A20 (A20), Ubiquitin Specific Peptidase 4 (USP4), Ubiquitin-Specific-Processing Protease CYLD (CYLD), TRAF Family Member Associated NF-*κ*B Activator (TANK), Tripartite Motif Containing Protein 38 (TRIM38), and Small Heterodimer Partner (SHP) [96–98]. TAK1 activation is inhibited by Tripartite Motif Containing 5 (TRIM5; TRIM30a in mouse) and A20 [99]. In addition to these signalling molecules, the transcription factor NF-*κ*B is suppressed by B-Cell Lymphoma 3-Encoded Protein (Bcl-3), NF-*κ*B Inhibitor Delta (IkBNS), Nuclear Receptor Related 1 (Nurr1), Activating Transcription Factor 3 (ATF3), and PDZ and LIM Domain Protein 2 (PDLIM2) [100].

**Figure 2:**
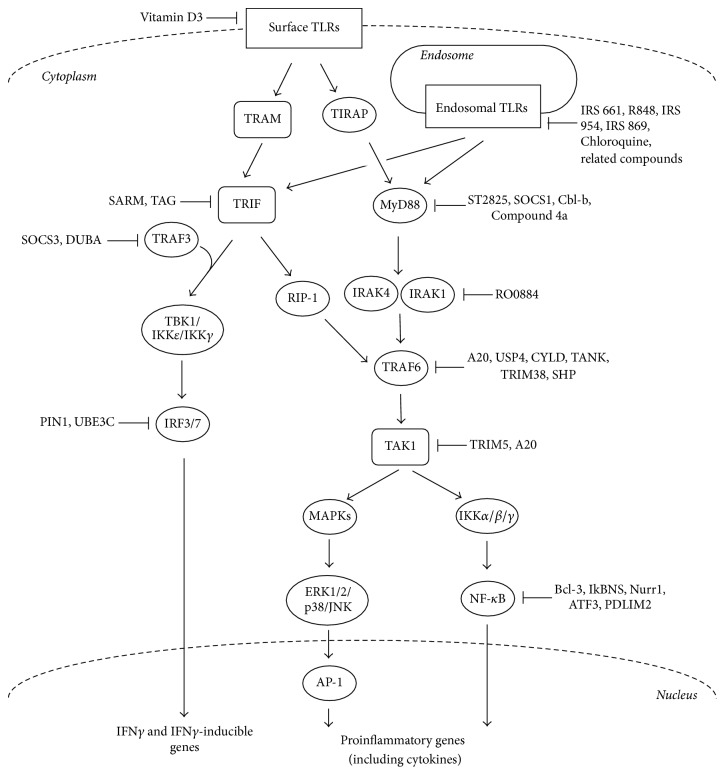
TLR signalling inhibitors. The regulation of TLR signalling has been achieved through the application of both natural and synthetic inhibitory molecules, which target each of the key molecules in the TLR signalling pathways, acting through a wide range of mechanisms. (AP-1, activator protein 1; ATF, activating transcription factor; ERK, extracellular signal-regulated kinase; IKK, inhibitor of kappa light polypeptide gene enhancer in B-cell kinase; IRAK, IL-1 receptor-associated kinase; JNK, c-Jun N-terminal kinase; MKK, MAPK kinase; RIP1, receptor interacting protein 1; TAB, transforming growth factor-b-activated kinase 1/MAP3K7-binding protein; TAK, transforming growth factor-activated kinase; TRAF, tumour necrosis factor receptor-associated factor); A20, Tumour Necrosis Factor Inducible Protein A20; ATF3, Activating Transcription Factor 3; Bcl-3, B-Cell Lymphoma 3-Encoded Protein; Cbl-b, E3 Ubiquitin Protein Ligase Cbl-b; CYLD, Ubiquitin-Specific-Processing Protease CYLD; DUBA, Deubiquitinating Enzyme A; HA900, high molecular weight hyaluronan; IkBNS, NF-*κ*B Inhibitor Delta; Nurr1, Nuclear Receptor Related 1; PDLIM2, PDZ and LIM Domain Protein 2; PIN1, Peptidylprolyl Cis/Trans Isomerase, NIMA-Interacting 1; SARM, Sterile Alpha and TIR Motif Containing 1; SHP, Small Heterodimer Partner; SOCS1, Suppressor of Cytokine Signalling 1; SOCS3, Suppressor of Cytokine Signalling 3; TAG, splice variant of the adaptor TRAM; TANK, TRAF Family Member Associated NF-*κ*B Activator; TRIM5, Tripartite Motif Containing 5; TRIM38, Tripartite Motif Containing Protein 38; UBE3C, Ubiquitin Protein Ligase E3C; USP4, Ubiquitin Specific Peptidase 4.

Activation of the TRIF-dependent pathway is suppressed by Sterile Alpha and TIR Motif Containing 1 (SARM) and a splice variant of the adaptor TRAM called TAG [101–103], both acting as direct TRIF inhibitors. TRAF3 activation is negatively regulated by Suppressor of Cytokine Signalling 3 (SOCS3) and Deubiquitinating Enzyme A (DUBA) [104]. SOCS3 is itself upregulated by high molecular weight hyaluronan (HA900), making it also a key molecule in regulating TRAF3 activation [105]. IRF3 activation is negatively regulated by Peptidylprolyl Cis/Trans Isomerase, NIMA-Interacting 1 (PIN1) and Ubiquitin Protein Ligase E3C (UBE3C or RAUL) [100]. (Figure 2).

At the RNA level, micro-RNAs (miRNAs) such as miR-146a, miR-199a, miR-155, miR-126, miR-21, miR-29, miR-148/152, and miR-466l regulate the stability of mRNAs that encode TLR signalling molecules [98]. Furthermore, the stability of mRNAs encoding cytokines are regulated by Regnase-1 and the zinc finger protein Tristetraprolin (TTP) [41, 98]. It was shown that, after being treated with a variety of TLR ligands, Regnase-1-deficient macrophages produce large amounts of cytokines and increased autoantibody production in Regnase-1-deficient mice [106].

One of the more interesting areas of research for therapeutic application of TLR modulators is the development of atypical, nonstimulatory DNA sequences called immune-regulatory DNA sequences (IRS) to regulate immune-stimulatory ligands of TLR7 and TLR9 as well as altogether inhibiting TLR7 and TLR9 stimulation [107]. Ligands of TLR9 include CpG-containing immune-stimulatory sequences (CpG-ISS) of diverse origin, including sequences of viral origin, CpG sequences which have been mutated, or mammalian telomere TTAGGG motif repeats [107–115].

A kind of TLR7 inhibitor (exemplified by IRS 661) acted specifically on mouse splenocytes to reduce their response to the TLR7 agonist R848, while having no effect on the response to a CpG-ISS, which is a TLR9 agonist. It also efficiently blocked antiribonucleoproteins (RNPs) but not anti-DNA-induced IFN*α*, indicating the role of TLR7 in this process. On the other hand, the TLR9 inhibitor IRS 869 was effective in inhibiting TLR9 activation by CpG-ISS, while having no effect when the TLR7 ligand R848 was used to activate the splenocytes, proving that anti-dsDNA-induced IFN*α* is TLR9-dependent. A newer type of inhibitor, exemplified by IRS 954 (derived by combining the three 5′ residues from the TLR7 inhibitor IRS 661 (TGC) with the TLR9 inhibitor IRS 869), could significantly inhibit splenocyte activation by ligands to TLR7 and TLR9 [50]. This IRS 954 was shown to strongly inhibit IFN*α* production by pDCs in response to ICs containing DNA or RNA. R848, through its mechanism of being a TLR7 agonist, was also found to induce production of IFN-*γ* in invariant NKT cells, which can alleviate allergic inflammation [116].

TLR modulators and inhibitors offer great promise with high selectivity and effectiveness when tested out in animal model for most autoimmune diseases. However it is important to recall when attempting to test inhibitors targeting TLRs and their signalling pathways for therapeutic applications using disease models in mice that humans and mice have different expression and distribution of TLRs and that any responses produced are just as different; for example, in humans, treatment with a TLR9 ligand produces a response dominated by the IFN*α* pathway [117–119] (stimulating numerous IFN-regulated genes elevated in lupus [120, 121]), while in mice TLR9 activation leads to the expression of proinflammatory cytokines such as IL-6, IL-12, or TNF-*α* via macrophage and myeloid DC activation.

## 8. Conclusion

TLRs have been shown to cover a much greater recognition repertoire than originally thought and a number of autoimmune diseases are either initiated or aggravated by TLR dysregulation. Thus further work in the field will definitely focus on uncovering more pieces to this ever growing signalling puzzle providing researchers and clinicians a clearer picture of the actual signalling pathways and viable, effective, druggable targets.

In fact a number of clinical trials are currently underway. These include antagonists of TLR4, such as TLR4 targeted monoclonal antibody NI-0101, by Novimmune, which has several potential applications including asthma and rheumatoid arthritis and is the first antibody for TLR4 to pass Phase I clinical trials for safety and tolerability [122]. Antagonists of the endosomal TLRs (specifically TLR7, TLR8, and TLR9) are also being studied, such as IRS954 by Dynavax and IMO3100 by Idera, who have published results from their Phase II trials for this drug [123], amongst others. As indicated by such clinical trials, the inhibition of aberrant TLR signalling induced by endogenous DAMPs or exogenous PAMPs has high potential as a viable therapeutic approach in humans for the treatment of autoimmune diseases.

The insight from such TLR signalling research should also direct the medical field to rethink the classification of some diseases or the way in which they are treated. Over the past decade lots of new components and interactions have been discovered and it is plausible to postulate that there is much more to be discovered. This being said, the position of the classification, development, and treatment of complexes or multifactorial diseases should also be reconsidered to include elements of autoimmunity.

Moreover, we are now starting to gain the ability to modulate specific TLR activity, which should be used to the benefit of patients suffering from such TLR-dependent autoimmune diseases. In order to achieve this goal, more studies on the inhibitors mentioned above and others are required in order to provide alternatives to the current limited therapeutic options, providing a long-term solution, with reduced off-target effects.

## Figures and Tables

**Table 1:** TLRs involved in autoimmune disease with the ligands causing the immune reaction. (EDN: eosinophil-derived neurotoxin; Hsp: heat shock protein; HMGB1: high-mobility group box 1 protein.)

TLRs	Potential endogenous ligands
TLR2	HSP60, HSP70, HSP90 fragments, hyaluronic acid, versican, HMGB1, biglycan, EDN

TLR3	mRNA, dsRNA

TLR4	HSP22, HSP60, HSP70, HSP72, HSP90, HMGB1, oxidised phospholipids, heparin sulfate, fibronectin, tenascin-C, *β*-defensin 2, versican, hyaluronic acid, minimally modified-low-density lipoprotein, fibrinogen, lung surfactant protein A

TLR7	U1snRNP RNA, ssRNA

TLR8	ssRNA

TLR9	Hypomethylated CpG-DNA
